# GO-Enabled Bacterial Cellulose Membranes by Multistep, In Situ Loading: Effect of Bacterial Strain and Loading Pattern on Nanocomposite Properties

**DOI:** 10.3390/ma16031296

**Published:** 2023-02-02

**Authors:** Tobiasz Gabryś, Beata Fryczkowska, Urška Jančič, Janja Trček, Selestina Gorgieva

**Affiliations:** 1 Department of Material Engineering, Faculty of Materials, Civil and Environmental Engineering, University of Bielsko-Biala, ul. Willowa 2, 43-309 Bielsko-Biala, Poland; 2 Department of Environmental Protection and Engineering, Faculty of Materials, Civil and Environmental Engineering, University of Bielsko-Biala, ul. Willowa 2, 43-309 Bielsko-Biala, Poland; 3 Institute of Engineering Materials and Design, Faculty of Mechanical Engineering, University of Maribor, Smetanova ul. 17, 2000 Maribor, Slovenia; 4 Department of Biology, Faculty of Natural Sciences and Mathematics, University of Maribor, Koroška Cesta 160, 2000 Maribor, Slovenia

**Keywords:** bacterial cellulose, graphene oxide, nanocomposite, structural analysis

## Abstract

This paper presents the results of research on the preparation and properties of GO/BC nanocomposite from bacterial cellulose (BC) modified with graphene oxide (GO) using the in situ method. Two bacterial strains were used for the biosynthesis of the BC: *Komagataeibacter intermedius* LMG 18909 and *Komagataeibacter sucrofermentans* LMG 18788. A simple biosynthesis method was developed, where GO water dispersion was added to reinforced acetic acid-ethanol (RAE) medium at concentrations of 10 ppm, 25 ppm, and 50 ppm at 24 h and 48 h intervals. As a result, a GO/BC nanocomposite membrane was obtained, characterized by tensile strength greater by 150% as compared with the pure BC (~ 50 MPa) and lower volume resistivity of ~4 ∙ 10^9^ Ω × cm. Moreover, GO addition increases membrane thickness up to ~10% and affects higher mass production, especially with low GO concentration. All of this may indicate the possibility of using GO/BC membranes in fuel cell applications.

## 1. Introduction

Cellulose is a widely available biopolymer synthesized mainly by plants, as well as fungi, protozoa, and prokaryotes [1]. Chemically, it is a linear homopolysaccharide composed of 3000–14,000 D-glucopyranose molecules linked with *β*-1,4-glycosidic bonds [2]. Plant-derived cellulose also contains other biopolymers, such as hemicelluloses and lignins. These compounds can be removed using chemical treatment and purification methods before further processing [3,4].

A fascinating pure form of cellulose is bacterial cellulose (BC). It is composed of linear *β*-1,4-glucan chains forming protofibrils, which combine into nanofibrils to form a compact, three-dimensional network [5]. The diameter of a single BC fibril does not exceed 100 nm and the product obtained in this bioreaction is in the form of a flat membrane [6,7]. One of the ways to synthesize BC is acetic fermentation, carried out in the presence of microorganisms, including gram-positive and gram-negative bacteria [8,9,10,11,12].

BC possess a relatively large specific surface area, high porosity, high flexibility, and mechanical strength (GPa-scale), as well as a high degree of crystallinity, reaching as far as 80% [13,14,15]. An exceptional feature of the BC is its ability to absorb, store, and desorb large amounts of water (more than 200 times its dry weight). This property is closely related to the structure of BC. Another very important feature of BC is biodegradability by many microorganisms, although not by the human body [16]. Moreover, it is a durable and environmentally friendly product. BC is applied in pharmacology and biomedicine [2,17,18,19,20] and can also be used in textiles, cosmetics, and food products [21,22]. In addition, the potential applications of BC include air purification, water treatment, food storage or energy conversion, electronic paper, audio membrane, and so on [22,23,24].

In order to give the BC membrane new, unique properties, it can be modified using methods such as ex situ, in situ, or solvent dissolution-regeneration [11]. The most important criterion differentiating these methods is that the ex situ method introduces additives onto the finished BC product. The literature describes many examples of the preparation of BC composites; e.g., with silk fibroin, proteins, gelatin, silver nanoparticles, zinc oxide, hydroxyapatite, or graphene oxide (GO) [11,14,25,26,27]. The in situ modification method introduces additives or nanoparticles that are soluble in water or form dispersion with it, directly into the BC culture medium used at the beginning of biosynthesis. In this one-step process, nanoadditives intertwine with the emerging network of BC nanofibers [11,14,28,29]. The literature describes the possibility of obtaining composites based on BC membranes with the addition of electrically conductive polymers as well as graphene and GO [13,29,30,31]. The third method of BC modification is solvent dissolution-regeneration. It involves dissolving BC; adding additives or nanoadditives, e.g., polypyrrole/carbon nanotubes or polyvinyl alcohol; and then precipitating the obtained composite [32,33].

An interesting and intensively researched nanoadditive is GO, a two-dimensional nanomaterial with different types of oxygen functional groups on its surface [34]. GO easily forms stable dispersions with water and other organic solvents, e.g., DMF and ethylene glycol [35,36]. The presence of many oxygen functional groups on the GO surface promotes the formation of dispersions with solutions of other polymers, which also have functional groups [37].

BC is undoubtedly such a polymer, featuring hydroxyl groups in its structure, thanks to which it can form hydrogen bonds with GO [38]. The methods of obtaining BC nanocomposites with GO are particularly well known. The literature describes a GO-modified BC nanocomposite, which can potentially be applied to remove impurities [29,31]. Other researchers have described a method of obtaining a multi-layer and durable BC/GO composite material, where GO was applied on the surface of the forming BC film [38]. In turn, Urbina et al. reported the in situ method to obtain bacterial cellulose-graphene oxide spherical nanocarriers where the GO was directly added to the medium [14]. Moreover, GO has been introduced into the BC network to prepare conductive nano papers as an innovative energy storage device [13,39]. The use of carbon nano additives can improve the properties of the nanocomposites such as porosity and strength and provide it with electro-conductive properties. Therefore, improving these parameters, which in the case of biomaterials are particularly important, increases the application capabilities. All of this allows for the potential use of such a nanocomposite as an adsorbent, scaffold, or dressing material [40,41,42,43]. Carbon-related nanomaterials can be used as biomaterials in tissue engineering and drugs carriers [14,44].

This paper presents research results about BC membranes modified with GO using a multistep, in situ loading method. This method was chosen to solve a critical challenge—GO agglomeration in the BC production medium. Two acetic acid bacterial strains were used to produce BC and GO/BC nanocomposites—*Komagataeibacter intermedius* and *Komagataeibacter sucrofermentans*. Their effect and loading pattern on GO/BC nanocomposite properties were studied to obtain homogeneous novel membranes that take advantage of the outstanding properties of BC and GO, including improved resistivity—an important property in fuel cell membrane application. The work is a continuation of our previous article, differing in that specific strains of bacteria were used and research was carried out confirming the possibilities of practical application, including mechanical and physico-chemical tests [45].

## 2. Materials and Methods

### 2.1. Chemicals

The reagents used for the preparation of RAE medium and purification of obtained BC were as follows: D-glucose (≥99%), peptone from meat enzymatic digest, and sodium hydroxide (≥97%, pellets) purchased from Sigma Aldrich (Darmstadt, Germany); yeast extract and micro agar purchased from Duchefa Biochemie (Haarlem, The Netherlands); disodium hydrogen phosphate dihydrate purchased from Merck (Darmstadt, Germany); citric acid purchased from Caleo (Graz, Austria); and acetic acid (99.8%) and ethanol (96%) purchased from Honeywell (Charlotte, NC, USA). The reagents for the production GO included the following: graphite powder < 20 μm purchased from Sigma-Aldrich, potassium permanganate (≥99%), and sulfuric acid (98%) and hydrogen peroxide (30%) purchased from Avantor Performance Materials Poland S.A. (Gliwice, Poland). GO synthesis description and testing of its properties (XRD, DSC, FTIR) were described in our earlier work [46].

### 2.2. Microorganisms

*Komagataeibacter intermedius* LMG 18909 and *Komagataeibacter sucrofermentans* LMG 18788 were maintained and precultured in the Laboratory of Microbiology, Department of Biology, Faculty of Natural Sciences and Mathematics, University of Maribor.

### 2.3. Medium

The RAE medium for bacterial cellulose production was selected on the basis of our experience and literature data [47]. RAE media were used for both types of bacteria (*K. intermedius* and *K. sucrofermentans*). Liquid RAE media consisted of glucose (40 g/L), peptone from meat (10 g/L), yeast extract (10 g/L), Na_2_HPO_4_ × 2H_2_O (3.38 g/L), citric acid (1.37 g/L), acetic acid (10 mL/L), and ethanol (10 mL/L). Solid RAE media for the bacteria revitalization and inoculation process consisted of the same ingredients as well as agar (10 g/L). Before use, all of the media were autoclaved at 121 °C for 20 min with the glucose solution autoclaved separately. Moreover, acetic acid and ethanol were added to the medium after the autoclaving process. The pH of the media was 4.1.

### 2.4. Growth Conditions

The bioprocesses were performed in 250 mL Erlenmeyer flasks equipped with a membrane screw cup. The flask containing 50 mL RAE media was inoculated with a single bacterial colony and incubated in a water bath for 7 days at 30 °C. For the first 24 h, the flasks were agitated by linear shaking at 120 rpm; after this, the shaking was discontinued and the bioprocess continued under static conditions. In this way, samples of reference bacterial cellulose were obtained, referred to as iBC (in the case of *K. intermedius*) and sBC (in the case of *K. sucrofermentans*).

**Figure 1:**
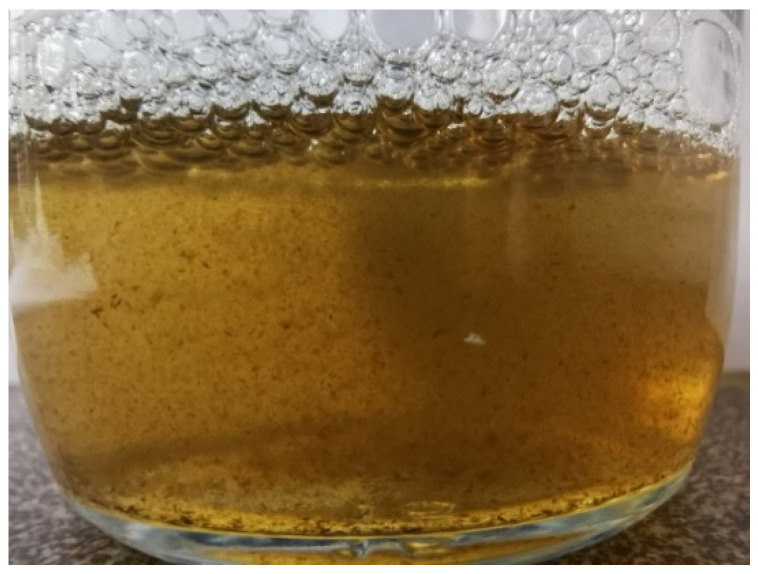
Visible aggregates of GO in RAE liquid medium (at pH = 4.1).

In order to synthesize the GO/BC nanocomposite, GO water dispersion with a concentration of 10 ppm, 25 ppm, and 50 ppm was added to the RAE liquid medium. Owing to the chemical structure of GO and its possible reduction above 80 °C, the dispersion was not autoclaved. Unfortunately, already at the first dose (3.33 mL and 5 mL) in contact with the RAE medium, the GO nanoparticles aggregated and sedimented (Figure 1).

**Figure 2:**
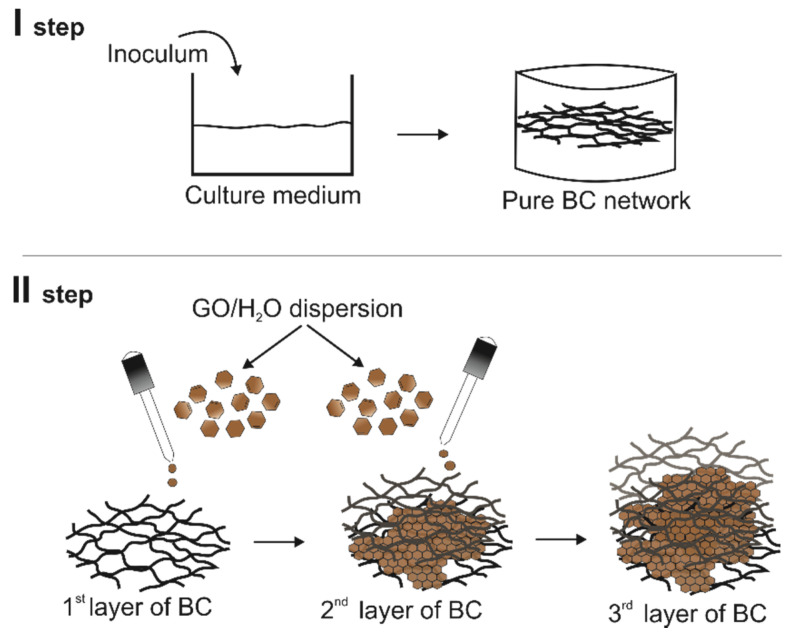
The loading process of GO/H_2_O application onto the BC network surface [45].

Therefore, it was decided to introduce the GO nanoadditive into the BC culture in small portions. In the GO/BC nanocomposite, GO water dispersion with a concentration of 10 ppm, 25 ppm, and 50 ppm was applied to the surface of the generated BC network in two ways. The first consisted of applying 5 mL of GO dispersion with a given concentration after 48 h and 96 h, while the second consisted of applying 3.33 mL of GO dispersion at the given concentration after 48 h, 72 h, and 96 h. In both cases, in the end, the amount of dispersion applied was the same, with only the volume of doses and time intervals being variable. Thus, a layer-by-layer nanocomposite material with GO and BC was obtained with two replications for each treatment. Figure 2 shows the process of applying GO dispersion on the BC surface, similar to our previous publication [45].

The designations of pure BC samples and GO/BC nanocomposite are summarized in Table 1.

### 2.5. Purification Process

**Figure 3:**
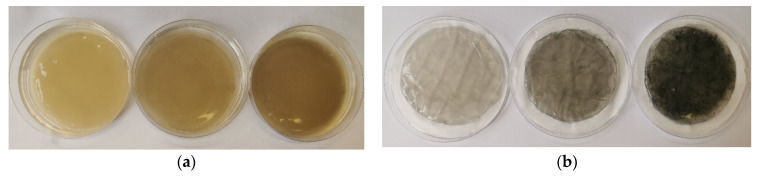
Examples of GO/BC nanocomposite samples with 10 ppm, 25 ppm, and 50 ppm (from left to right) GO concentration, before (**a**) and after (**b**) the neutralization process in 0.5 M NaOH.

After incubation, the BC and GO/BC nanocomposites were taken from the bioprocess and rinsed with MiliQ water to remove any residual media. Then, each GO/BC nanocomposite was placed in a beaker with 100 mL of 0.5 M NaOH. The purification process was carried out in a water bath at 80 °C for 1 h with a linear shaking speed of 70 rpm. Then, bioreaction products were washed several times with MiliQ water until a neutral pH was obtained. Figure 3 presents sample photos of GO/BC nanocomposites (from the left: sBC_10/3, sBC_25/3, sBC_50/3) before and after the neutralization process.

### 2.6. ζ Potential Analysis of GO Dispersions

The ζ potential measurements were performed to determine the stability of the GO dispersion in the liquid culture medium. For this purpose, Litesizer 500 (Anton Paar, Graz, Austria) with a unique Omega measuring cuvette was used. The measurement was taken in a thermostated cell at a temperature of 25 °C using Milli-Q water. The ζ potential was measured in a pH range from 2 to 12, being adjusted by NaOH (0.01 M) and HCl (0.01 M).

### 2.7. Physicochemical Properties

Initially, the GO/BC membranes obtained in the experiment were dried and then weighed on a Sartorius analytical balance with an accuracy of 0.0001 g to determine the weight of individual samples.

The thickness of the dry GO/BC nanocomposites was measured using a digital micrometer (Inside, 3109 Series, Zamudio, Spain). Before measurements, the GO/BC nanocomposites were conditioned for 24 h at room temperature. Each value is the average of four measurements made randomly along each of the GO/BC nanocomposites.

The water contact angle (CA) measurements were carried out using an OCA 35 optical device (DataPhysic Instruments GmbH, Filderstadt, Germany) equipped with a video measuring system with an optical camera and a high-performance table adapter. The volume of the Milli-Q water droplet was 3 μL. All measurements were performed at room temperature, in triplicates, with average and standard deviation reported.

The tensile modulus (MPa), tensile strength (MPa), and elasticity (%) of the GO/BC nanocomposite were determined using Shimadzu, AG-X plus 10 kN electromechanical universal testing machine. Dry GO/BC nanocomposite samples with specimen dimensions of 10 mm × 20 mm were mounted vertically. The effective clamping distance was 25 mm. The application of tensile force (10 kN load cell) proceeded at 1 mm min^−1^. Three or five specimens were tested per sample and average values and standard deviations were calculated.

**Figure 4:**
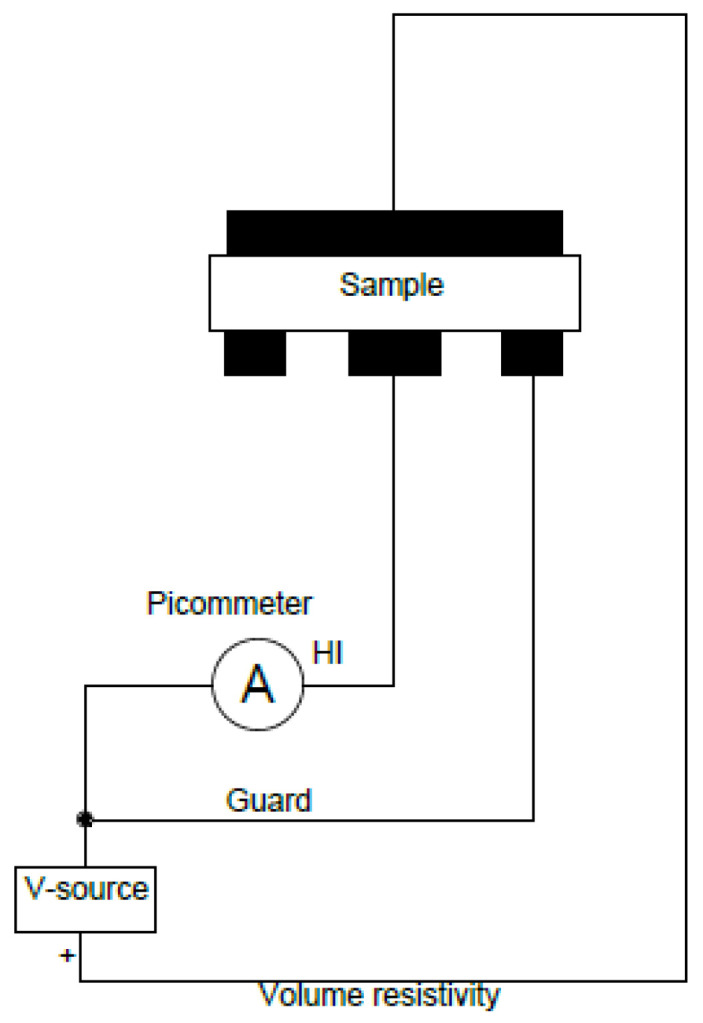
Diagram of the volume resistivity measurement technique.

The measurements of the volume resistivity of the obtained GO/BC nanocomposite were carried out in accordance with ASTM D275 standard using the Keithley meter model 6517A (Cleveland, OH, USA) and the Keithely test chamber, model 8009 (Cleveland, OH, USA). Samples of the test material were placed in a measuring cell between the electrode system. The measurement was carried out at a voltage of 50 V DC during an electrification time of 10 s. For each sample, five measurements were taken, from which an average value was determined. A measurement was also carried out for a reference sample without the addition of GO to demonstrate the effect of this additive on the electrical properties of the test material. A diagram showing the volume resistivity measurement method is shown in Figure 4.

The volume resistivity was measured by applying a voltage potential to opposite sides of the sample, measuring the resulting current flowing through the sample and then converting according to the following formula:

(1) $$\rho =\frac{V\times 22.9}{I\times t}\left[\Omega \times cm\right]$$

where *ρ* is the volume resistivity of the sample; *V* is the voltage applied; *I* is the measured current; *t* is the mean thickness of the sample expressed in centimeters; and 22.9 is the constant characteristic of the electrode geometry.

### 2.8. Structural Analysis

GO/BC nanocomposite surface observation was carried out using a high-resolution Phenom ProX scanning electron microscope (SEM) from Thermo Fisher Scientific (Pik Instruments, Piaseczno, Poland), operating at 10 kV. The samples were previously coated with a 20 nm gold layer using a Leica EM ACE 200 low-vacuum coater (Wetzlar, Germany).

The chemical structure of GO/BC nanocomposite materials with different GO concentrations was analyzed using a Spectrum GX FTIR spectrometer (PerkinElmer, Waltham, MA, USA) with a Golden Gate ATR attachment and a diamond crystal. The transmission spectra were obtained within the range of 4000–650 cm^−1^, with 16 scans and a resolution of 4 cm^−1^. Reference samples (iBC and sBC) were scanned in parallel. All scans were performed at room temperature.

Wide angle X-ray scattering (WAXS) studies were performed using a D2 Phaser diffractometer (Bruker AXS GmbH, Karlsruhe, Germany) using the Bragg–Brentano reflection geometry method. CuKα radiation (λ = 1.54 Å) was emitted at an accelerating voltage of 30 kV and an anode current of 10 mA. A scintillation counter was used as a detector. The tests were carried out in the range of 2θ from 5° to 60° in steps of 0.03° and acquisition time of 0.25 s per one step. Before the measurements, a small piece of material was cut, placed on a quartz crystal holder, and measured at room temperature.

Thermogravimetric investigations were performed using a TA Instruments Q500 Thermogravimetric Analyzer (New Castle, DE, USA). The measurements were conducted in a nitrogen atmosphere (flow 60 mL/min), in the temperature range from 30 to 500 °C, at a heating rate of 20°/min. TG and DTG curves were analyzed using Universal V2.6D TA Instruments software (New Castle, DE, USA).

### 2.9. Statistical Methods

The results of the study were subjected to statistical analysis consisting of the application of Student’s *t*-test for independent paired samples at a 5% level of significance.

## 3. Results and Discussion

### 3.1. ζ Potential of GO Dispersion and Stability of GO in RAE Medium

**Figure 5:**
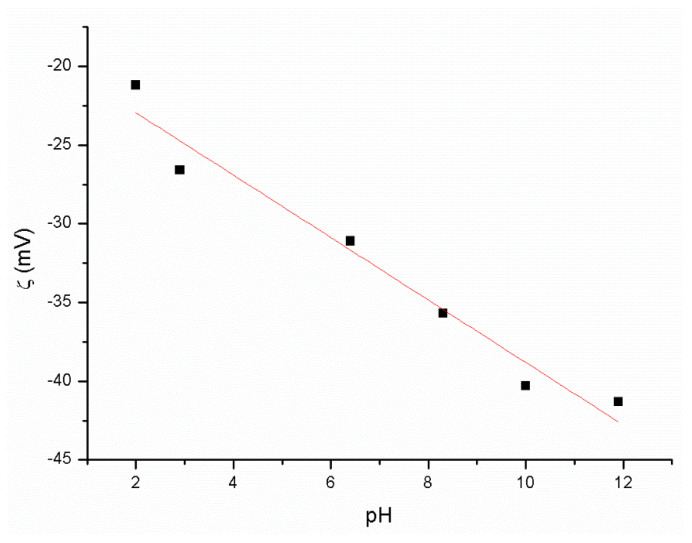
Zeta potential (ζ) of GO/H_2_O dispersion in pH dependency.

Because of the fact that GO is in the form of nanosheets, its stability in liquid dispersions is an essential parameter as it affects its distribution pattern within the final GO/BC nanocomposites to which it has been added. Therefore, before starting the experiment, it was necessary to explore how GO would behave within the culture medium upon its application to BC. The liquid RAE medium used has a pH of about 4.1. Therefore, ζ = f (pH) was measured in a wide range of pH from 2 to 12 (Figure 5), which covers the pH of interest.

The analysis showed (Figure 5) that the ζ potential of the GO water dispersion is about −27 mV at pH 4.1, which indicates its stability under these conditions [48]. A team of other researchers obtained almost identical results. Kartic et al. confirmed in his work that the zeta potential for GO at the same pH value ranges from −50 mV to −20 mV and, for a pH of 4.1, the zeta potential was ~−30 mV [49]. However, research on the water dispersion of GO and on its dispersion in a complex environment such as the RAE medium differs significantly. Therefore, despite the stability of GO at pH 4.1 in the water environment, at the time of contact with RAE, the GO medium underwent immediate aggregation and sedimentation (Figure 1). The research results obtained by us differ from those described in the literature, which reports that the addition of GO dispersion to the liquid medium did not cause its aggregation [38]. This may result from using a different medium, e.g., Hestrin–Scharmann (HS) [14,28]. The reason behind aggregation is a high presence of ions in the RAE medium, causing extensive charge screening. Because of this, the diffuse electric double layer around the particle surfaces is thinner, causing van der Waals force to dominate the interparticle interaction [50]. This is according to the classical theory of dispersion stability developed by Derjaguin, Landau, Verwey, and Overbeek (DLVO) [51].

As the aggregation state strongly depends on the time window investigated [50], we assume that spreading GO dispersion on already formed BC in a few intervals will hinder the aggregation owing to the limited time for contact among the particles.

### 3.2. General Characteristics of the Obtained Material

**Figure 6:**
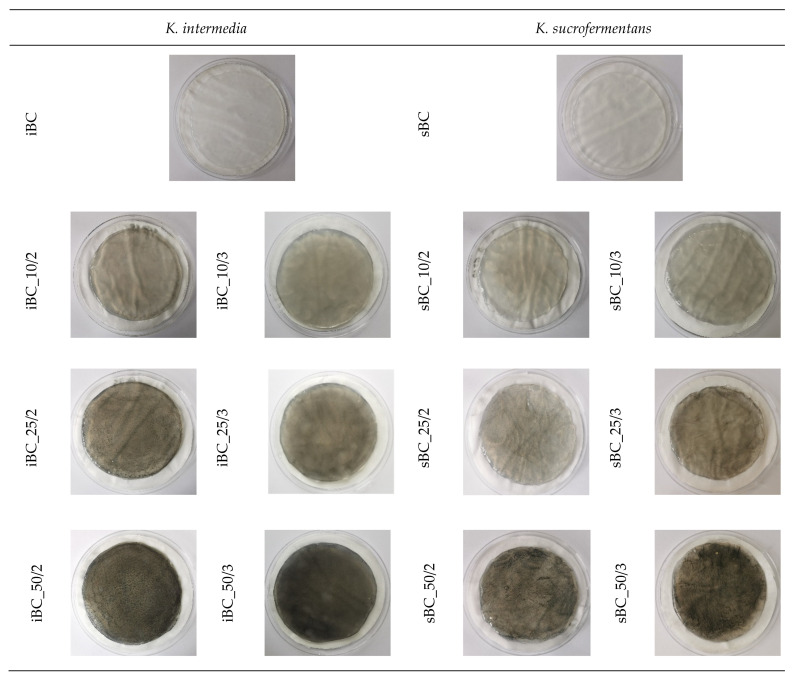
Photos of obtained pure BC and GO/BC nanocomposites.

As a result of the experiment, pure BC and a library of GO/BC nanocomposite were obtained, photos of which are presented in Figure 6. The photographs show a change in color along with the amount of nanoadditive introduced. The reference samples (iBC and sBC) are colourless and almost identical to the naked eye. As the concentration of GO used increases (10, 25, and 50 ppm, respectively), the colour of the samples becomes darker. For samples sBC_50/2 and sBC_50/3, individual GO aggregation sites are visible as darker points.

In order to characterize the obtained pure BC and GO/BC nanocomposites, the following measurements were carried out: production yield; thickness; FTIR; XRD; contact angle; SEM; and mechanical, thermal (TG, dTG), and electrical properties.

**Figure 7:**
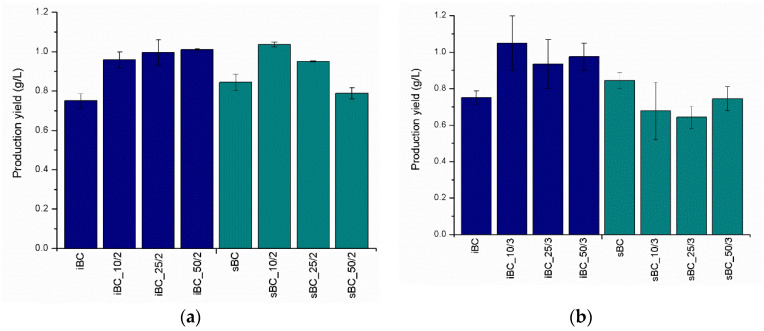
Production yield (g/L) of dry BC and GO/BC nanocomposites with (**a**) two and (**b**) three GO loading steps.

Production yield measured from obtained dry material (Figure 7) indicates that *K. sucrofermentans* is a better producer of BC than *K. intermedius* in the absence of additives. Under the same conditions, it produced 12% more BC dry matter. An interesting fact is that, in the case of *K. intermedius*, regardless of the GO loading time intervals, a trend of increasing the mass of the GO/BC nanocomposites with an increase in the concentration of GO dispersion is observed. It ranges from 24% (iBC_25/3) to 39% (iBC_10/3) as compared with the iBC control sample without the addition of GO. In the case of *K. sucrofermentans*, this relationship looks different and is more closely related to the GO loading methodology, i.e., frequency and single dose. Applying GO at intervals of 48/24/24 h, as described in Table 1, results in a light decrease trend in the mass of the obtained nanocomposite, with an increase in the concentration of GO. A similar effect occurs with samples sBC_10/2, sBC_25/2, and sBC_50/2.

The analysis of the thickness of dry GO/BC nanocomposites (Table 2) shows mainly the differences in the thickness of the obtained nanocomposite depending on the strain used. The use of *K. intermedius* results in an initial BC production of about a 20% thicker membrane than in the case of the *K. sucrofermentans* strain. Thus, the difference between the average thickness values of the iBC and sBC samples is statistically significant. This phenomenon is observed for both pure BC (iBC and sBC) and the ones doped two and three times with GO. The addition of GO during BC synthesis was shown an impact on the thickness of all GO/BC nanocomposite membranes. The highest increases in BC mass are observed in the sBC_10/2 sample and amount to as much as 38%. Thus, the results we obtained confirm that the created reaction conditions favor the biosynthesis of BC.

### 3.3. Structural Analysis

**Figure 8:**
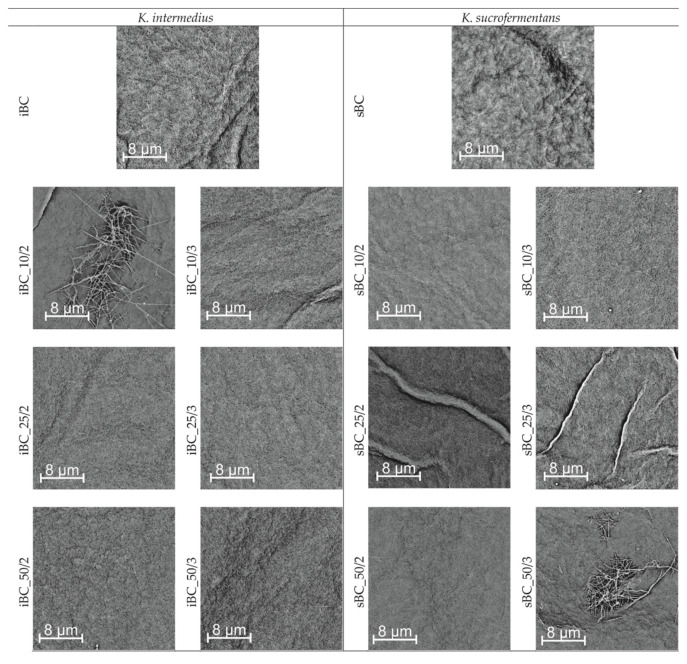
SEM microphotographs of all nanofiber membranes obtained using both bacterial strains. Magnification: 100,000×.

The SEM images (Figure 8) show that GO/BC nanocomposites are made of nanofibers to form a compact 3D tight network, where individual fibrils are randomly distributed. The SEM analysis does not show GO nanosheets on the surface of GO/BC nanocomposites. This may indicate that GO is entangled between nanofibers in deeper layers. This may result from the method of applying GO to the surface of the forming nanofiber network, with individual layers being arranged alternately, and the first and last layer formed by BC. In addition, the microphotographs show no residues of bacterial cells, which proves their thorough removal during the neutralization process in NaOH. In addition, the analysis of the microphotographs indicates the existence of characteristic differences between pure bacterial cellulose synthesized by individual strains. The sBC sample, unlike iBC, shows a more compact, less porous structure, where the nanofibers adhere closely to each other. In iBC, on the other hand, it is possible to easily distinguish individual nanofibers from the rest of the cellulose matrix.

**Figure 9:**
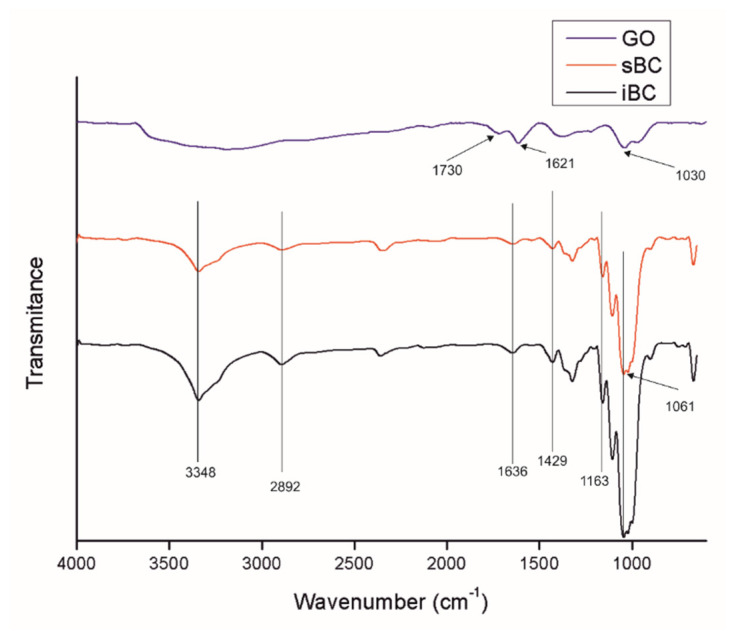
FTIR spectra of GO and neat BC produced by *K. intermedius* and *K. sucrofermentans*. The spectra range from 4000 cm^−1^ to 650 cm^−1^.

The FTIR spectrographs of GO (Figure 9) show the presence of peaks at 3000–3600, 1730, 1621, and 1030 cm^−1^, which correspond to a very broad adsorption of -OH groups, two most characteristic peaks corresponding to the stretching vibrations of carboxyl C=O, -C=C- (stretching mode of sp^2^ network), and C-O-C groups, respectively [52]. BC shows characteristic FTIR peaks for cellulose type I at 3348, 2892, 1636, 1429, and 1061 cm^−1^, corresponding to the hydroxyl (-OH) stretching vibrations, -CH asymmetric stretching, hydroxyl (-OH) bending vibrations, CH_2_ symmetric bending, and bond of glycosidic bridges (C-O-C and C-O skeletal stretching) [14,28].

**Figure 10:**
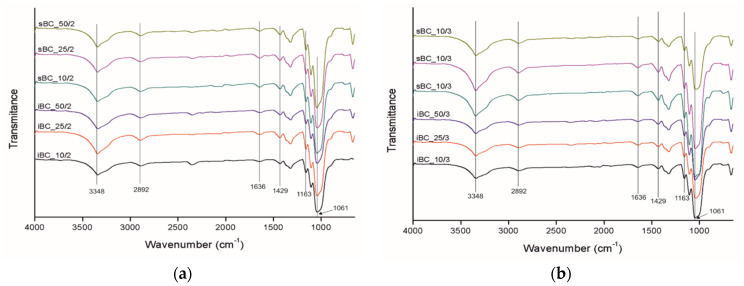
FTIR spectra of GO/BC membranes produced by *K. intermedius* and *K. sucrofermentans* with different levels of GO addition with two loadings (**a**) and three loadings (**b**). The spectra range from 4000 cm^−1^ to 650 cm^−1^.

In the case of GO/BC nanocomposite membranes (Figure 10), the characteristic BC peaks appear in the same location without big differences (Figure 9). In these samples, no peaks characteristic of GO can be seen, which may be caused by a too low concentration and strong dispersion of GO nanoparticles in the cellulose matrix with no new peaks found. The peak in some samples, the maximum of which is 2350 cm^−1^, is derived from CO_2_ in the atmospheric air.

**Figure 11:**
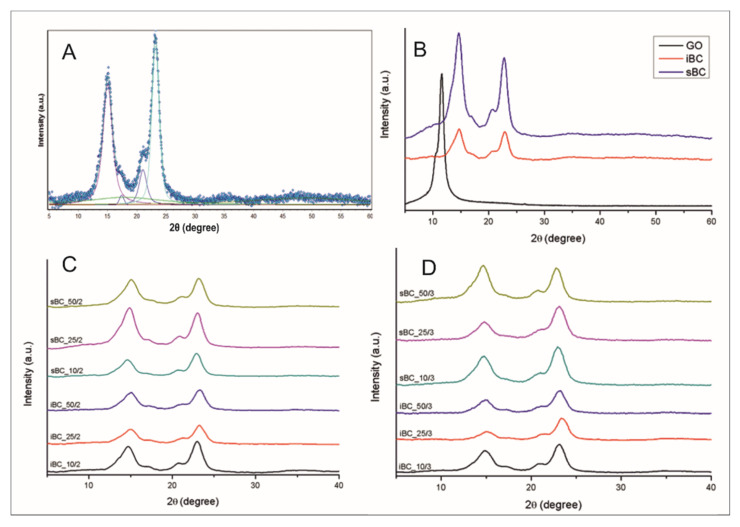
Distribution of XRD pattern for sBC sample into crystalline and amorphous components (**A**), XRD patterns of GO and neat BC produced by *K. intermedius* and *K. sucrofermentans* (**B**), and XRD patterns of GO/BC nanocomposites with different levels of GO addition with two loadings (**C**) and three loadings (**D**).

The crystallinity index (CI) of obtained materials was determined based on WAXS analysis by the peak deconvolution method [53]. For this purpose, each WAXS pattern was distributed into individual crystalline and amorphous components using the WaxsFit software [54]. In this software, deconvolution is performed by means of an approximation method. It consists of the construction of a theoretical curve, which is composed of functions related to individual crystalline peaks and amorphous maxima. The shape of each peak was approximated using a linear combination of the Gaussian and Cauchy’s functions. The parameters of these functions are found through the best fitting of the theoretical curve to the experimental one using a suitable optimization procedure. The theoretical curve was fitted to the experimental data using the Rosenbrock’s double-criteria optimization method described by Rabiej et al. [55]. The crystallinity index was determined as the ratio of the sum of the surface areas under the crystalline peaks to the total area under the scattering curve. An example of the distribution of bacterial cellulose XRD diffraction pattern into crystalline and amorphous components using this software is shown in Figure 11A.

Analysis of X-ray curves of GO/BC nanocomposite (Figure 11C,D) as well as reference samples (Figure 11B) clearly indicates the presence of characteristic peaks that are easy to identify. In the case of BC membranes (Figure 10b), there are four characteristic crystalline peaks for cellulose I, where 2θ equals 14.6°, 16.8°, 20.6°, and 22.8°, corresponding to crystal planes (1 (−1) 0), (110), and (200), respectively [38,53]. The peak for 2θ at 20.6° is the result of the overlapping scattering coming from two planes (012) and (102). It occurs when crystallites are not oriented in the sample relative to the crystallographic axis c. Scattering from these planes is weak and often not registered, and even poor orientation eliminates it [56].

XRD curves for GO/BC nanocomposites also indicate the presence of these peaks without significant shifts compared with reference samples made of pure BC. This indicates that the crystalline structure of BC has not changed after the introduction of GO into the cellulose matrix. In addition, it was found that, in the GO/BC nanocomposite XRD curves, regardless of the GO concentration, there is no GO-specific peak for 2θ at 11°. This may indicate the good distribution of GO nanoparticles throughout the volume of the cellulose matrix [14,38,57]. The analysis of the degree of crystallinity indicates that the addition of GO does decrease the cryatallinity index (Table 3). Dhar et al. in their work described a similar correlation but with rGO, whereby a higher concentration of rGO decreased the crystallinity index by up to 5% [28]. Rashidian et al. came to similar conclusions, which in turn showed that the GO addition reduced CI by 10 percentage points compared with neat BC [58]. This is because of all kind of additives, especially nanoadditives, impede the formation process of BC nanofibrils, which form highly ordered crystalline areas [59].

**Figure 12:**
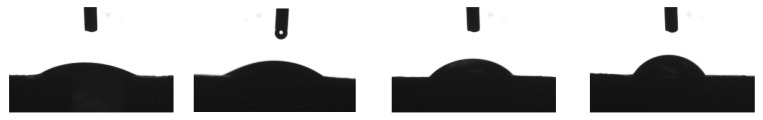
Illustrative microphotographs taken during the contact angle measurement. From the left, sBC, sBC_10/2, sBC_25/2, and sBC_50/2.

**Figure 13:**
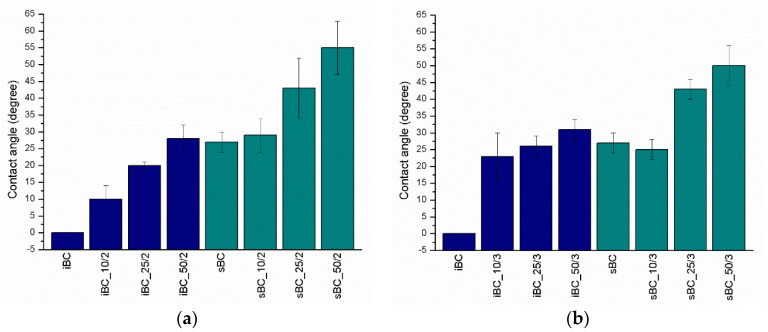
Contact angle of BC and GO/BC nanocomposite with two GO loadings (**a**) and three GO loadings (**b**).

The surface hydrophilicity of obtained BC and GO/BC nanocomposites was determined by measuring the contact angle (Figure 12). The water contact angle values show an increasing trend linked with GO concentration in all GO/BC nanocomposites (Figure 13). This means that GO addition decreases the hydrophilicity of GO/BC nanocomposites. A similar dependence was demonstrated by research studies of Urbina et al., where the swelling ratio of water was lower along with the increase in GO content. Further, this is known to be strictly related to the hydrophilicity of the material surface [14]. The explanation of this fact by Huang et al. may be that GO might not have been homogeneously dispersed in the cellulose matrix and that GO tended to aggregate [60]. It is worth noting that all nanocomposites obtained using *K. sucrofermentans* are characterized by significantly higher contact angle values as compared with those obtained with *K. intermedius*. In the case of the iBC sample, it was impossible to measure CA because, when the drop was applied to the surface of the membrane, the drop was immediately absorbed by the material. However, all obtained BC and GO/BC nanocomposites demonstrate hydrophilicity, with their contact angle being lower than 90°. Exemplary microphotographs taken during the contact angle measurement are shown in Figure 12.

A statistical test was carried out comparing the average values of the contact angle in terms of the number of GO doses and in terms of the bacterial strain used. Samples were tested sequentially: iBC_10/2; iBC_10/3; iBC_50/2; iBC_50/3; sBC_10/2; sBC_10/3; sBC_50/2; sBC_50/3. Hypothesis H_0_ assumes that, at 5% significance, the difference between the means of the samples is statistically insignificant. The test showed that, for a given level of significance, the difference between the mean values is statistically significant except for the pair iBC_10/3 and sBC_10/3, where |U| < u_α_. This means that both the selection of the strain and GO do have an impact on the CA value.

The mechanical properties of the obtained nanofiber membranes are presented in Table 4. Significant differences between the reference samples and those containing GO are noticeable. The tensile strength values for the iBC and sBC samples were 19.41 ± 1.2 and 17.34 ± 4.2 MPa, respectively. The results of the statistical analysis comparing iBC and sBC samples in terms of mechanical parameters (tensile strength, tensile modulus, and elongation) at the significance level of 5% showed that, in terms of the first criterion, the difference between the means of the samples is not significant, i.e., |U| < in α, while in terms of the other two parameters, these samples differ significantly, where |U| > u_α_, which means that the type of bacterial strain under specific conditions affects these parameters. Interesting conclusions can be drawn based on the results for the samples containing the smallest addition of GO (iBC_10/3 and sBC_10/2), for which the tensile strength values increased more than threefold and were 60.9 MPa and 51.51 MPa, respectively, which proves that the effect of GO on material strength is positive. This phenomenon is linked to the interaction between GO and the cellulose matrix like hydrogen bond formation [38,58]. Moreover, the 3D structure of the GO network can improve this parameter. The addition of GO at higher concentrations, during both two- and three-fold loading, resulted in a gradual decrease in breaking strength, which, however, was still 1.5 times higher as compared with the samples without the addition of GO. Considering the fact that GO, like all nanoadditives, shows the ability to agglomerate, in the cellulose matrix as well, it can be assumed that this was the cause of material weakening at higher concentrations (25 ppm and 50 ppm) and a decrease in strength.

**Figure 14:**
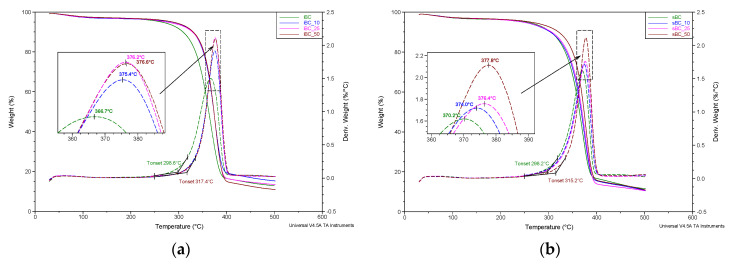
TG and DTG curves of the studied *K. intermedius* (**a**) and *K. sucrofermentans* (**b**) bacterial cellulose samples with different levels of GO addition.

Thermogravimetric analysis (Figure 14) shows that the thermal degradation process of the obtained material is a single step and occurs from approximately 250 to 400 °C. The temperature at the beginning of the degradation process (based on DTG curves) is the lowest for the iBC membrane (298.6 °C) and the highest for the iBC_50 membrane (317.4 °C). The temperature of the maximum thermal decomposition is also the lowest for the reference sample iBC (366.7 °C) and increases monotonically together with the content of the GO nanoadditive up to 376.6 °C for the iBC_50 membrane. For sBC membranes, the temperature of the initial thermal degradation point is the lowest for the sBC sample (298.2 °C) and the highest for the sample with the maximum GO content: sBC_50 (315.2 °C). Moreover, in this case, the monotonicity of the decomposition process is preserved, which is manifested by an increase in the degradation temperature of more than 7 °C (sample sBC_50) in relation to the reference sample without the addition of GO (370.2 °C). It follows that GO causes a significant shift at the extrapolated temperature of the beginning of the decomposition and the actual temperature of thermal degradation. A similar relationship has been observed by us in the case of GO/CEL fibers and described in another paper [60,61].

The analysis of the volume resistivity results obtained for BC and GO/BC nanocomposites (Table 5) shows the differences in resistivity depending on the amount of the nanoadditive introduced and the method of its incorporation. The values range from 10^11^ Ω × cm for pure cellulose reference samples to 10^9^ Ω × cm (iBC_50/2 and sBC_50/2). The results show a trend of decreasing resistivity with an increase in GO content in the material. Despite the fact that GO is a carbon nanoadditive, it conducts electricity poorly. This is because of the presence of a large number of carbon groups with sp^3^ hybridization. Nevertheless, its presence in the cellulose matrix of the obtained GO/BC membranes reduces the volume resistivity by two orders of magnitude. The greatest influence of GO on the change in resistivity of the obtained material was observed for the series of samples subjected to double BC loading in GO dispersion, for both *K. intermedius* and *K. sucrofermentans strains.* This may result from the fact that the single dose of loading, which in this case was 5 mL (Table 1), aggregated on the neat BC surface and was not evenly absorbed into the formed network of cellulose nanofibers. As a result, local areas containing more GO reduce the volume resistivity.

## 4. Conclusions

This paper presents research results on BC membranes modified with GO using a multistep, in situ loading method. This method was chosen with the aim of solving a critical challenge—agglomeration of GO in the medium used for BC production. Two acetic acid bacterial strains were used to produce BC and BC-GO nanocomposites—*Komagataeibacter intermedius* and *Komagataeibacter sucrofermentans*. The obtained GO/BC nanocomposite material was characterized in terms of structural and physicochemical aspects. The results of the study indicate a significant influence of GO on the properties of GO/BC nanocomposites, including a decrease in volume resistivity by two orders of magnitude (iBC_50/2 ~4.4 × 10^9^). WAXS analysis as well as thermal analysis (TG, DTG) demonstrated interactions between BC and GO, which are visible in a decrease in the degree of crystallinity. In addition, as a result of the addition of GO to the cellulose matrix, a monotonic increase in the thermostability of the material was noted, significantly increasing the temperature of thermal decomposition from 366.7 °C (iBC) to 376.2 °C (iBC_50).

## Figures and Tables

**Table 1:** Designation of the obtained BC and GO/BC nanocomposites.

*K. intermedia*	*K. sucrofermentans*	GO Dispersion Concentration [ppm]	GO Loading Dose	Intervals of Loadings [hour]
iBC	sBC	-	-	-
iBC_10/2	sBC_10/2	10	2 × 5.0 mL	
iBC_25/2	sBC_25/2	25		48/48
iBC_50/2	sBC_50/2	50		
iBC_10/3	sBC_10/3	10	3 × 3.33 mL	
iBC_25/3	sBC_25/3	25		48/24/24
iBC_50/3	sBC_50/3	50		

**Table 2:** Thickness of dry material from pure BC and GO/BC nanocomposites.

Thickness of Material after Drying [μm]			
iBC	23.3 ± 1.2	sBC	18.0 ± 0.9
iBC_10/2	26.6 ± 7.4	sBC_10/2	24.8 ± 7.7
iBC_25/2	23.6 ± 6.7	sBC_25/2	23.4 ± 7.1
iBC_50/2	26.1 ± 6.4	sBC_50/2	17.9 ± 3.3
iBC_10/3	25.0 ± 4.2	sBC_10/3	18.7 ± 3.3
iBC_25/3	23.1 ± 5.1	sBC_25/3	19.7 ± 5.7
iBC_50/3	23.5 ± 4.2	sBC_50/3	20.5 ± 3.4

**Table 3:** Crystallinity index (%) of obtained BC and GO/BC nanocomposites.

Crystallinity Index (CI) [%]			
iBC	70.9	sBC	64.8
iBC_10/2	70.1	sBC_10/2	65.7
iBC_25/2	69.6	sBC_25/2	65.3
iBC_50/2	69.5	sBC_50/2	63.0
iBC_10/3	68.4	sBC_10/3	67.3
iBC_25/3	66.9	sBC_25/3	66.0
iBC_50/3	65.1	sBC_50/3	65.9

**Table 4:** Mechanical properties of BC and GO/BC nanocomposites.

Sample	Tensile Strength (MPa)	Tensile Modulus (MPa)	Elongation (%)
iBC	19.41 ± 1.2	1614 ± 29	1.46 ± 0.17
iBC_10/2	39.22 ± 0.87	1115 ± 127	1.65 ± 0.17
iBC_25/2	55.84 ± 1.47	2231 ± 175	2.56 ± 0.63
iBC_50/2	30.01 ± 2.69	2301 ± 171	1.86 ± 0.26
iBC_10/3	60.93 ± 10.51	2508 ± 271	2.59 ± 0.40
iBC_25/3	45.38 ± 11.17	1252 ± 247	3.17 ± 0.69
iBC_50/3	43.02 ± 13.31	3401 ± 490	1.69 ± 0.35
sBC	17.34 ± 4.2	1426 ± 24	0.69 ± 0.29
sBC_10/2	51.51 ± 7.01	2586 ± 72	1.49 ± 0.29
sBC_25/2	48.16 ± 3.51	1529 ± 172	2.33 ± 0.82
sBC_50/2	22.21 ± 1.52	2344 ± 324	0.95 ± 0.16
sBC_10/3	42.20 ± 11.49	1405 ± 287	1.15 ± 0.54
sBC_25/3	38.78 ± 2.38	2714 ± 546	2.05 ± 0.36
sBC_50/3	38.44 ± 4.06	1917 ± 396	2.59 ± 0.31

**Table 5:** Volume resistivity of the obtained BC and GO/BC nanocomposites.

Volume Resistivity (Ω × cm)			
*K. intermedius*		*K. sucrofermentans*	
iBC	3.5 × 10 ^11^	sBC	5.5 × 10 ^11^
iBC_10/2	8.7 × 10 ^10^	sBC_10/2	3.9 × 10 ^11^
iBC_25/2	1.4 × 10 ^10^	sBC_25/2	9.7 × 10 ^10^
iBC_50/2	4.4 × 10 ^9^	sBC_50/2	7.5 × 10 ^9^
iBC_10/3	7.7 × 10 ^10^	sBC_10/3	2.5 × 10 ^11^
iBC_25/3	6.5 × 10 ^10^	sBC_25/3	2.0 × 10 ^11^
iBC_50/3	6.1 × 10 ^10^	sBC_50/3	8.8 × 10 ^10^

## Data Availability

Data available upon request from the authors.
